# Impact of Individual and Situational Factors on Employees' Unethical Pro‐Organizational Behavior: Based on an FsQCA Approach

**DOI:** 10.1002/pchj.70066

**Published:** 2025-11-18

**Authors:** Zhuojie Li, Xiaozhan Wang, Leiru Wei, Paweł Jurek

**Affiliations:** ^1^ Institute of Psychology University of Gdansk Gdansk Poland; ^2^ School of Economics and Management Zhengzhou University of Light Industry Zhengzhou China

**Keywords:** configurational approach, fuzzy set qualitative comparative analysis, unethical pro‐organizational behavior

## Abstract

Despite the exploration of the impact of individual and external situational factors on Unethical Pro‐Organizational Behaviors (UPB) and its boundaries to some extent, existing research has not sufficiently delved into the complementary relationships and the interactive effects among multiple factors, making it challenging to fully elucidate the complexity of UPB outcomes. Drawing upon prior research on UPB, this study employed the fuzzy set qualitative comparative analysis (fsQCA) method, integrating configuration theory and individual‐context interaction theory, to gather a total of 550 datasets from seven Chinese food enterprises and a professional research platform (Credamo). The findings revealed that no single factor was essential for UPB; instead, the five factors encompassing individual psychological and external situational aspects coexist in multiple configurations, resulting in three distinct driving mechanisms. Furthermore, there exists a causal asymmetry within the driving mechanisms of UPB. Based on these insights, it is imperative to adopt a differentiated management approach from a holistic perspective, considering the specific context, fostering an ethical and supportive organizational climate, being vigilant about the potential adverse impacts of transformational leadership, and guiding employees with a high inclination toward Machiavellianism.

## 1 Introduction

It is commonly accepted that unethical behavior refers to actions that harm others, violate laws, or contravene universal moral standards, such as deception and cheating. Because these behaviors disrupt social order and result in potential harm to society, it is one of the hot topics to understand their formation mechanisms in order to prevent and reduce such behaviors. Previously, the research results suggest that unethical behaviors are self‐centered and emerge at the expense of organizations or others (Klein and Epley 2017). However, sometimes employees may intentionally engage in actions that violate ethical standards and behavioral norms, but with the intention of benefiting the organization or others. Umphress et al. (2010) referred to these prosocial‐oriented unethical behaviors as unethical pro‐organizational behaviors (UPB), which can promote effective functions of organizations or their members while intentionally violating core social values, moral customs, laws, and proper behavioral norms (Umphress and Bingham 2011). This type of “good intentions lead to bad results” behavior is not uncommon in management practices too. For instance, senior engineers at Volkswagen developed a “defeat device” designed to cheat on emission tests, resulting in the company having to pay over $15 billion in fines in 2016. The head of Yuwell Medical (China) inflated the prices of oximeters to increase the company's profits, which led to a fine of 2.7 million RMB for seriously jeopardizing the legitimate rights and interests of consumers in 2023. These unethical behaviors of employees, which undermine organizational culture and employee motivation, harm the interests of consumers, defame the reputation of companies, and may further lead to legal risks and talent loss. As such, they have become an important issue for the long‐term strategic development of companies. Therefore, creating a suitable circumstance to reduce the occurrence of such behaviors is of great importance.

Regarding the significant research topic of the antecedents of UPB, the academic community has conducted a series of valuable exploratory studies. Existing literature has primarily analyzed the antecedent factors of UPB from two distinct levels: the organizational level and the individual level. At the organizational level, the factors involved encompass aspects such as the ethical climate, leadership styles, and high‐performance work systems (Graham et al. 2015; Kim et al. 2023; Miao et al. 2013; Yan and Zeng 2018; Y. Zhang et al. 2017). On the individual level, key elements include psychological entitlement, Machiavellianism, affective commitment, organizational identification, and moral disengagement (Castille et al. 2018; Dadaboyev et al. 2024; Mishra et al. 2022; Yurtkoru and Ebrahimi 2017). Umphress and Bingham (2011) clearly pointed out that UPB is the outcome of the interaction between individual factors and situational factors. Nevertheless, current academic research on the interaction among the antecedent factors of UPB is still in its initial stage, and the underlying mechanism by which situational factors and individual factors jointly influence employees' UPB remains to be further clarified and understood in depth. In light of this, to comprehensively reveal the triggering mechanism of employees' UPB, it is essential to systematically explore the specific action paths of multiple interacting antecedent conditions on employees' UPB and further elaborate on the substitution effects among these antecedent conditions. In contrast to traditional quantitative research, which places emphasis on revealing the “net effect” between variables, the fuzzy‐set Qualitative Comparative Analysis (fsQCA) method enables a comprehensive consideration by incorporating multiple antecedent variables into configurations (Pappas and Woodside 2021). Through meticulous configuration analysis, it delves deep into the influence paths of combinations of antecedent variables on the outcome variable and further accurately identifies the substitution effects among variables.

Based on the above background, this study first incorporates individual factors and situational factors into the construction of configurations, thus forming configurations of antecedent conditions that influence employees' UPB. Subsequently, by applying the fsQCA method and conducting rigorous configuration analysis, this study endeavors to reveal the path mechanism that triggers employees' UPB. Additionally, through comparative analysis of different configurations, it aims to accurately identify the substitution relationships among different antecedent conditions or combinations of conditions. This research aims to further enrich the theoretical research system in the field of UPB by deeply analyzing the internal mechanism that triggers employees' UPB, and it also intends to provide practical references and guidance for relevant management practices.

## 2 Literature Review

### 2.1 Unethical Pro‐Organizational Behavior

UPB, an inherently contradictory concept extending from unethical behavior, combines the characteristics of being pro‐organizational and unethical. It represents a complex combination of organizational citizenship behavior and unethical conduct (Umphress and Bingham 2011). On one hand, the motivational aspect of UPB, which is intended to safeguard the interests of the organization or its members, constitutes the most significant distinction between UPB and general unethical behavior. On the other hand, the essence of UPB lies in its unethical nature, as it represents a form of immoral conduct. Currently, scholars have explored the antecedents of UPB from multiple perspectives. Existing theoretical research primarily draws on four theoretical frameworks, namely the social cognitive theory, social exchange theory, social identity theory, and social learning theory. These theories have provided valuable insights into understanding the factors contributing to the emergence of UPB, enabling a more comprehensive exploration of this complex phenomenon within the organizational context.

(1) Social identification theory. Tajfel (1986) proposed social identity theory, which suggests social identity mainly stems from group membership or eligibility. People actively pursue and maintain positive social identity to enhance self‐esteem. Studies show employees with high organization identification—the psychological bond between an individual and their organization—tend to internalize the organization's successes and failures as personal outcomes. This internalization drives them to align with the organization's expectations and interests, potentially leading to pro‐organizational behaviors. Notably, even when such actions are unethical, individuals may engage in UPB to preserve organizational belonging (M. Chen et al. 2016). Therefore, organizational identification is widely recognized as a critical antecedent of UPB, with empirical studies consistently demonstrating a positive correlation. For instance, Holmes and Howard (2023) argued that organizational identification exhibits a double‐edged sword effect: employees with strong identification are more likely to engage in both ethical courageous actions and unethical behaviors for the organization. Li (2024) further validated this relationship through meta‐analysis, revealing cultural dimensions (individualism, power distance, masculinity, uncertainty avoidance, and indulgence) moderate the association. Beyond this, social identity theory also elucidates other antecedents of UPB: transformational leadership, characterized by inspiring and motivating followers, is linked to UPB (Khushk et al. 2022; Yan and Zeng 2018), with Zhang and He (2024) finding organizational commitment partially mediating this relationship. Additionally, job embeddedness—the extent to which an individual is connected to their work environment—has been identified as a contributing factor. Ghosh (2017) and Lee et al. (2022) proposed that deeply embedded employees may resort to UPB as a strategy to enhance or maintain their status within the organization.

(2) Social cognitive theory. The social cognitive theory holds that unethical behavior occurs partly because cognitive mechanisms weaken the constraints of moral norms on their own behavior (Bandura 1991). Moral disengagement explains this justificatory mechanism at the cognitive level and provides a theoretical foundation for the research on UPB in the organizational context. According to social cognitive theory, most people have personal moral standards for self‐regulation and supervision—normal self‐monitoring prevents unethical behavior and triggers guilt, but damaged self‐monitoring weakens moral constraints, leading to unethical acts (Bandura et al. 1996). Existing research shows external factors such as psychological entitlement, performance pressure, inclusive leadership, abusive supervision, and bottom‐line thinking directly or indirectly drive UPB through moral disengagement (Yurtkoru and Ebrahimi 2017; Zhang, Zhou, et al. 2024). For example, Chen and Liang (2017) noted employees under high‐performance task pressure who activate moral disengagement may use unethical means to achieve their performance goals, harming society or groups outside the organization.

(3) Social exchange theory. Rooted in the premise that human behavior is inherently driven by reciprocal exchanges, social exchange theory posits that individuals act to maintain mutually beneficial relationships. Umphress and Bingham (2011) argued that employees may resort to UPB as a form of repayment to their organization or employer, sustaining positive social exchange. Specifically, a robust social exchange relationship fosters trust and commitment, which in turn can influence the likelihood of UPB (S. Zhang and Yang 2024). Numerous factors linked to UPB have been identified under this framework. For instance, perceived organizational support has been shown to predict UPB (Chhabra and Srivastava 2023). Similarly, the leader‐member exchange (LMX) relationship plays a crucial role: Bryant and Merritt (2021) and Kelebek and Alniacik (2022) demonstrated that high‐quality LMX interactions can prompt employees to engage in behaviors benefiting the organization, even if unethical. Other influential elements include affective commitment (Yurtkoru and Ebrahimi 2017), empowering leadership (S. Zhang and He 2024), and high‐commitment human resource management practices (Luu 2023; Uymaz and Arslan 2022). Tang et al. (2023) proposed that the interaction between employees' perceived performance pressure and leader‐member exchange increases employees' willingness to engage in UPB. Moreover, Zhang and Yang (2024) empirically verified that workplace friendship and emotional commitment positively correlate with the occurrence of UPB, reinforcing the role of social exchange in shaping such behaviors.

(4) Social learning theory. Social learning theory explains UPB by emphasizing the interplay between the environment, individual characteristics, and learning processes—individuals acquire ethical/unethical behaviors through experience, observation, and others' action reinforcement. Environmental factors like organizational culture, norms and values shape employees' behavioral choices. Zhao and Zhou (2017) demonstrated that employees may perceive UPB as acceptable in corporate environments promoting false philanthropy. Zhou and Ran (2023) found an inverted U‐shaped relationship between perceived organizational politics and UPB. Notably, the traditional Chinese concept of Zhongyong—the principle of moderation—moderates this association, highlighting cultural factors' role in behavior regulation. Individual factors also play a pivotal role. Personal values and moral frameworks influence how individuals interpret ethical dilemmas and evaluate the acceptability of UPB. Azhar et al. (2024) illustrated that subordinates may mimic their leaders' UPB, rationalizing it as appropriate to prioritize organizational interests. It demonstrated the power of observational learning in justifying unethical actions for organizational gain.

Overall, social identity theory, social cognitive theory, and social exchange theory are most widely used to understand UPB. Social identity and social cognitive theory can explain the “pro‐organizational” aspect of UPB, while social exchange theory accounts for the “unethical” part. Most existing studies explore the relationship between a certain variable and UPB from a single theoretical perspective. However, in real management situations, the influencing factors of UPB are complex, and there may be complementarity or combinations of multiple factors. This complexity hinders a comprehensive interpretation of UPB. Therefore, enhancing the robustness of theory is crucial for a deeper understanding of UPB.

The individual‐situation interaction perspective is a comprehensive theoretical framework widely applied in psychology. It can systematically and dynamically explain various functions and development processes within the structure of individual psychology and behavior. According to this theory, the overall system of the individual and the situation consists of two subsystems. The individual subsystem includes factors like individual psychology (the perception‐cognition‐emotion system), biological aspects (gender differences, physiological maturity, etc.), and behaviors (short‐term stress responses, long‐term behavior changes). The situational subsystem encompasses the real physical environment, the social environment as a source of information and stimuli, and the cultural environment as a long‐term influencing and triggering factor for events. These two subsystems interact continuously, promoting the development of individual psychological, biological, behavioral, and other functions. Through summarizing existing research, we found that factors triggering UPB can be categorized into two core categories: individual factors (psychological entitlement, Machiavellianism, affective commitment, organizational identification, moral disengagement, etc.) and situational factors (ethical climate, leadership type, high‐performance work systems, etc.). Some studies have explored how the interaction between these two types of factors influences UPB from the individual‐situation interaction perspective (Chhabra and Srivastava 2023; Graham et al. 2015; Tian and Peterson 2016).

From the perspective of individual employees, based on the social identity theory, perception and attitude can directly influence the individual's behavioral tendency. Therefore, different perceptions and attitudes of individuals may induce or inhibit UPB. Organizational identification, as an important variable of perception and attitude, has a particularly close relationship with UPB. In addition, considering that individuals with different personality traits may exhibit diverse behaviors, in the field of organizational behavior, the “dark personality” and “socially aversive” trait of Machiavellianism have gradually attracted scholars' attention, especially in research on negative behaviors such as employees' unethical behaviors or deviant behaviors. In light of this, this study incorporates two individual factors, Machiavellianism and organizational identification, into the research on the antecedents of UPB. From the situational perspective, according to social identity theory, different leadership styles can affect employees' identification with the organization, thereby influencing UPB. Transformational leadership, a positive and effective leadership behavior, is generally believed to influence employees' attitudes by inspiring subordinates and demonstrating leadership charisma. This makes it easier for employees to develop identification with both the leader and the organization, and they may engage in UPB (Effelsberg et al. 2014). Additionally, when employees perceive a specific atmosphere within the organization and identify with it, they will categorize themselves as members of the organization, and then develop cognitive attitudes and behavioral decisions consistent with the organizational atmosphere. According to social cognitive theory, when employees perceive that the organization has set overly high‐performance goals, in addition to generating internal self‐motivation, they will also experience corresponding psychological pressure. Therefore, high performance expectations may lead employees to take actions that improve work efficiency but violate social moral norms and even harm other stakeholders to achieve the company's performance goals. Therefore, this study selects three situational factors: transformational leadership, high performance expectations, and benevolence ethical climate.

In summary, this study aims to elucidate, from the individual‐situation interaction perspective, the influence mechanisms of Machiavellianism, organizational identification, transformational leadership, high‐performance expectation, and benevolence ethical climate on employees' UPB. Additionally, it will further explore and explain the interaction and substitution effects among the antecedent variables.

### 2.2 Individual Factors: Machiavellianism and Organizational Identification

Machiavellianism is an important personality trait that reflects the extent to which an individual is willing or inclined to use any means to achieve their own goals (Greenbaum et al. 2017). It is characterized by a disregard for moral standards, a lack of trust in others, a strong desire for control, and an aspiration for success and status. Previous studies have already demonstrated that Machiavellianism has a significant positive impact on unethical behavior (Castille et al. 2018). Employees with high levels of Machiavellianism prefer dynamic environments and are capable of adjusting their strategies according to different situations. They hold utilitarian values of “using any means to achieve the end” and tend to employ any method to achieve their personal goals (Gao and Zhao 2014). Therefore, when employees with high Machiavellianism face organizational performance pressure, their stable utilitarian moral sense will cause them to instinctively respond in a way that is economically rational and in line with their own interests, even if such behavior is unethical (Castille et al. 2018; Guo and Chen 2021). In addition, the higher the level of Machiavellianism, the weaker employees' moral awareness (Belschak et al. 2015) and the higher their tolerance for unethical behavior (Greenbaum et al. 2017). Thus, influenced by the tendencies such as the weak moral awareness of Machiavellians, the self‐regulatory function of the moral mechanism will be further suppressed. At this time, employees with high levels of moral disengagement will be more “at ease” and “without a guilty conscience” in implementing UPB. Therefore, Machiavellianism is an important factor affecting the UPB of employees.

Organizational identification, as a specific form of social identification (Ashforth and Mael 1989), has been proven to bring favorable impacts to the organization, such as lower turnover intentions of employees (Cole and Bruch 2006) and higher job performance (Ashforth et al. 2008). However, excessive organizational identification has also been confirmed to induce individuals' unethical behaviors (Ashforth and Anand 2003). This is because employees with a high level of organizational identification will internalize the organization's collective values into their personal values, and then think and choose their behaviors more from the organization's perspective (Ashforth et al. 2008). Chen et al. (2016) have demonstrated that organizational identification has a positive impact on UPB through moral justification. Umphress et al. (2010) have also confirmed that individuals with a strong sense of organizational identification are more likely to engage in UPB, as organizational identification provides the motivation for participating in such behaviors. Thus, organizational identification serves as a critical factor in influencing UPB.

Comparatively, while Machiavellianism drives UPB mainly from an individual's self‐centered motivation, organizational identification does so through the psychological connection and value alignment with the organization. Moreover, in terms of long‐term effects, over‐identification with the organization may lead to employee burnout or ethical dilemmas when the organization's actions deviate from the individuals' internalized values. In the short term, it can boost UPB as employees strive to meet the organization's needs. Cross‐culturally, in individualist cultures, organizational identification might be less likely to lead to UPB as individuals may place more importance on their own moral compass than on strict organizational loyalty. In summary, both Machiavellianism and organizational identification are critical factors in influencing UPB.

### 2.3 Situational Factors: Transformational Leadership, High Performance Expectation and Benevolence Ethical Climate

Transformational leadership is a leadership style in which leaders establish a working environment of mutual understanding and support with employees through leadership charisma, inspiration, intellectual stimulation, and individualized consideration (Bass 1985). Transformational leaders typically communicate the organizational vision to employees, pay attention to the employees themselves, and stimulate their higher‐level needs. By doing so, they encourage employees to make every effort for the organizational interests, thus promoting high‐level performance (Effelsberg et al. 2014). This psychological connection leads to a high level of organizational commitment among employees. Employees will closely connect the survival of the organization with their personal success or failure. The stronger the employees' organizational commitment, the more likely they are to prioritize organizational interests, aiming to maximize organizational benefits and ignoring ethical requirements. Therefore, under the guidance of transformational leaders, the likelihood of employees engaging in UPB increases significantly (Graham et al. 2015; Yan and Zeng 2018). Consequently, transformational leadership is an important factor affecting the UPB of employees.

Previous studies have shown that high performance expectations can lead employees to experience significant psychological pressure, which may drive them to engage in unethical behavior to achieve goals (Tang et al. 2023). Tian and Peterson (2016) found that when employees feel more pressure from supervisors and colleagues, they are more likely to sacrifice personal values for organizational goals, increasing their willingness to engage in UPB. Chen and Liang (2017) empirically demonstrated a significant increase in employee willingness to engage in UPB when faced with high performance expectations. This process is mediated by moral disengagement and positively moderated by employees' perception of market competition. Chen and Chen (2023) further confirmed a general positive correlation between performance pressure and UPB, and Mo et al. (2023) argued that performance goal orientation also has a positive influence on UPB participation. Accordingly, high performance expectation is an important factor affecting employees' UPB.

Organizational ethical climate refers to employees' perceptions of what is considered ethical behavior in the organization and how ethical issues are resolved (Trevino 1986). Malloy and Agarwal (2001) argue it clarifies employees' cognitive perception of organizational values and guides ethical judgment. Studies show that it has a significant impact on employees' ethical behaviors and decision‐making (Zoghbi‐Manrique‐de‐Lara and Guerra‐Baez 2016) and relates to counterproductive work behaviors and deviant behaviors (such as destruction of organizational assets, time theft, workplace bullying, etc.) (Leung 2008). According to Victor and Cullen (1988), there are five types of organizational ethical climate, including benevolence, instrumental, rules, laws and regulations, and independence. Among them, benevolence ethical climate is a positive ethical climate in which organizations adhere to altruistic principles and care about the interests of each employee. While caring about their own and the organization's interests, organization members working in this climate also pay attention to the interests of other stakeholders (X. Li and Peng 2022). According to the social cognitive theory, employees will adjust their cognition based on their environment and take certain actions to conform to the environment. This also implies that when employees are in a benevolent ethical climate, they are likely to perceive the organization's concern (H. Zhang and Cao 2021), develop a strong sense of belonging to the organization, and ultimately engage in UPB (S. Zhang and Yang 2024). Zhang et al. (2017) have also verified that benevolence ethical climate has a positive impact on UPB through moral justification. The impact of a benevolent ethical climate on UPB may also interact with other factors. For instance, in an organization with high performance expectations, a benevolent ethical climate might mitigate the negative effects of performance pressure on employees' ethical decision‐making to some extent. However, if the performance pressure is extremely high, even in a benevolent ethical climate, some employees may still be tempted to engage in UPB. Consequently, the benevolence ethical climate is also a crucial influencing factor of UPB.

## 3 Research Conceptual Model

**FIGURE 1:**
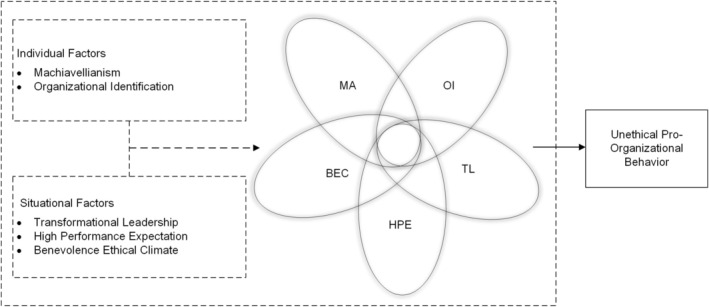
The conceptual model of outcome variables from two individual and three situational factors influencing UPB. BEC, benevolence ethical climate; HPE, high performance expectation; MA, Machiavellianism; OI, organizational identification; TL, transformational leadership; UPB, unethical pro‐organizational behaviors.

Based on configuration thinking, there is a synergistic effect between individual factors (Machiavellianism and organizational identification) and situational factors (transformational leadership, high performance expectations, and benevolence ethical climate) in predicting employees' UPB. In order to conceptualize the complex relationships between UPB and various antecedent variables, herein we propose a theoretical model (see Figure 1) to illustrate the interaction of five antecedent variables and the outcome variable. The individual and the situational factors are presented on the left side. The outcome variable, UPB is shown on the right side. The overlapping area in the middle represents the possible combinations among the variables, that is, the area where a certain variable may coexist with other variables.

## 4 Methodology

### 4.1 Research Design

Qualitative comparative analysis (QCA), developed by the Ragin group, adopts a holistic and systematic analytical approach. This approach is helpful for exploring how multiple antecedent conditions jointly contribute to the occurrence of an explained outcome. Therefore, it is highly suitable for addressing research questions characterized by conditionality and complexity in causality (Ragin 2008). In recent years, QCA has been widely applied in fields such as sociology and management (Fiss 2007; Pappas et al. 2016).

In this study, we adopted the QCA method for the first time to explore the various configurations of employee UPB for three reasons. Firstly, UPB results from multiple variables at different levels. These include personality traits and perceptions for individuals, leadership types, organizational policies and the organizational climate in the work situation. These variables exhibit complex causal relationships in these behaviors. Compared with traditional regression analysis, which focuses on analyzing the net effects of a single variable, the QCA method could be more suitable for exploring the complex concurrent relationships among diverse conditions and verifying the interaction effect of multiple variables on UPB (Rihoux and Ragin 2012). The QCA method's ability to explore complex concurrent relationships is thus highly relevant for understanding the multi‐faceted causes of UPB. Secondly, the configurations of conditions that lead to UPB are not unique in daily work situations. Using the QCA method, researchers can effectively identify the equifinality relationships between multiple condition variables and outcome variables (Meuer and Fiss 2020). This helps us to reveal different configurations of conditions that lead to UPB, which is crucial for comprehensively understanding the phenomenon of UPB. Thirdly, the QCA method can analyze the sufficiency and necessity of each antecedent factor for employee UPB from a set‐theoretic perspective. Conclusions derived from set relations have stronger theoretical and practical significance (Fiss 2007). Understanding the sufficiency and necessity of factors can provide more targeted suggestions for preventing and managing UPB in organizations.

Based on the type of data, QCA can be divided into crisp‐set qualitative comparative analysis (csQCA), multi‐value qualitative comparative analysis (mvQCA), and fuzzy set comparative analysis (fsQCA). Among them, fsQCA can be used to analyze both degree and category issues and can cover different types of condition data well. Given the complex nature of UPB, fsQCA's flexibility in handling different data types makes it an ideal choice for this study. It can capture the nuances in the relationships between antecedent conditions and UPB more effectively than other QCA types. Therefore, this study adopts the fsQCA method, and the selected data analysis software is fsQCA 4.1.

### 4.2 Sample and Data Sources

This study primarily collected data via a questionnaire survey. Based on the pre‐distributed questionnaire, some ambiguous or problematic items were revised to develop the final questionnaire. The questionnaire consists of six scales, with a total of 50 items. Except for the measure of UPB, five‐point Likert scales were used for all antecedent variables. All measures were collected concurrently with the demographic information, including participants' age, gender, education, tenure, position, etc.

UPB was assessed using the six‐item scale developed by Umphress et al. (2010). The scale included items such as “If the company needs it, I can cheat to protect the company's image.” Responses were measured on a seven‐point Likert scale (1 = *strongly disagree*, 7 = *strongly agree*), and the scale had a Cronbach's α of 0.93.

Machiavellianism was assessed with the 16‐item scale developed by Dahling et al. (2009). Sample items included “I'm willing to be unethical if it helps me get ahead.” Responses were rated on a five‐point Likert scale (1 = *strongly disagree*, 5 = *strongly agree*), and the scale's Cronbach's *α* was 0.90.

Organizational identification was evaluated using the six‐item scale developed by Mael and Ashforth (1992). Items included “When someone criticizes my company, it feels like criticizing myself.” Responses were measured on a five‐point Likert scale (1 = *strongly disagree*, 5 = *strongly agree*), and the scale's Cronbach's *α* was 0.85.

Transformational leadership was assessed with the eight‐item scale developed by Chen et al. (2006). Items included “My superior tries to make his subordinates happy.” A five‐point Likert scale (1 = *strongly disagree*, 5 = *strongly agree*) was used for responses, and the scale had a Cronbach's *α* of 0.85.

High‐performance expectation was assessed with the three‐item scale developed by Podsakoff et al. (1990). Items included “My superiors have high performance standards for employees.” Responses were rated on a five‐point Likert scale (1 = *strongly disagree*, 5 = *strongly agree*), and the scale had a Cronbach's *α* of 0.77.

Benevolence ethical climate was assessed using the five‐item scale developed by Liu and Jing (2010). Items included “In our company, the employees all take care of each other.” Responses were measured on a five‐point Likert scale (1 = *strongly disagree*, 5 = *strongly agree*), and the scale had a Cronbach's *α* of 0.83.

To ensure the relevance of the survey subjects to the research topic, data were collected through the following two main channels. First, we contacted relevant personnel of 7 leading enterprises in the food industry in Henan Province, China (including Shuanghui, Haoxiangni, Weilong, Mixue, Synear, Sanquan, and Baixiang Food) and distributed questionnaires to their employees via email. Second, we distributed questionnaires through the professional research company Credamo, targeting employees. After excluding invalid questionnaires with highly consistent answers and short completion times, 550 valid questionnaires were obtained, with an effective response rate of 91.7%. Among them, 123 questionnaires were collected offline and 427 were collected online.

A Mann–Whitney *U* test was conducted on the samples from these two channels. Since there was no significant difference between the two groups of samples, they were combined into one dataset for analysis. Among the respondents with valid data, 39.2% were male, and 60.8% were female; 89.6% of the respondents were aged between 25 and 44; 89.4% had a bachelor's degree or above; 23.3% of the respondents were from state‐owned enterprises, and 64.7% were from private enterprises; 52.6% of the respondents had been working in their current company for more than 5 years; and 88.2% of the respondents held middle‐level positions in their companies.

### 4.3 Reliability and Validity Testing

Data analysis of the collected valid samples was conducted using SPSS 23.0 and AMOS 24. As shown in Table 1, the Cronbach's *α* values of each variable were all above 0.7, and the CR values were all above 0.777, indicating good internal consistency of the scales and high overall questionnaire reliability. The KMO values for each latent variable exceeded 0.696, indicating that the research scales were suitable for factor analysis. The validity of the scales was tested using standardized factor loadings and average variance extracted (AVE). It was found that most of the standardized factor loadings for each item were above 0.6, and the Machiavellianism factor had 7 loadings above 0.6. The AVE values ranged from 0.360 to 0.705. This indicates that the scales have good construct validity and are suitable for further analysis.

**TABLE 1:** Measurement properties of variables.

Variables	No. of items	Cronbach's *α*	KMO	CR	AVE
UPB	6	0.933	0.919	0.935	0.705
MA	16	0.904	0.907	0.896	0.360
OI	6	0.845	0.866	0.851	0.490
TL	8	0.852	0.901	0.857	0.431
HPE	3	0.774	0.696	0.777	0.538
BEC	5	0.825	0.828	0.826	0.492

Abbreviations: AVE, Average Variance Extracted; BEC, benevolence ethical climate; CR, composite reliability; KMO, Kaiser‐Meyer‐Olkin. HPE, high performance expectation; MA, Machiavellianism; OI, organizational identification; TL, transformational leadership; UPB, unethical pro‐organizational behaviors.

### 4.4 Homologous Variance Testing

Given that all items of the scale are completed by the same respondent, this may introduce a certain degree of bias into the results. To address this concern, a homologous variance test was conducted using Harman's single‐factor test method. The analysis revealed that the characteristic root of the first factor was 13.12, with a variance contribution rate of 29.82%, which is less than half of the cumulative variance contribution rate (64.19%). Consequently, it can be concluded that the sample data does not exhibit significant common method bias.

### 4.5 Data Calibration

Before conducting configurational analysis using fsQCA, it is necessary to calibrate the variables to transform them into sets ranging from 0 to 1, in order to determine the degree to which the sample belongs to each set (Ragin 2008). Based on the characteristics of the sample data and in accordance with the mainstream practices in the academic community, this study takes the 90th and 10th percentiles of the sample data as the qualitative anchor points for “full‐membership” and “non‐membership”, respectively, and uses the 50th percentile as the cross‐point for calibration (Dou and Sun 2024; Pappas and Woodside 2021). The Calibrate function in the fsQCA 4.1 software was used to initially calibrate the data for each antecedent variable. Then, to improve the model performance, the calibrated values were uniformly increased by 0.001 to avoid generating fuzzy membership degrees of 0.5. The anchor points and descriptions of the outcome variable and antecedent variables in this study are shown in Table 2.

**TABLE 2:** Calibration rules and descriptive statistics of variables.

Variable	Calibration rules			Descriptive statistics			
	FM	CP	FNM	*M*	SD	Min	Max
MA	3.31	2.31	1.50	2.37	0.73	1.00	5.00
OI	3.00	1.67	1.33	1.97	0.72	1.00	5.00
TL	2.63	1.63	1.38	1.88	0.60	1.00	5.00
HPE	3.00	1.67	1.33	1.94	0.73	1.00	4.67
BEC	3.40	1.80	1.40	2.10	0.77	1.00	4.80
UPB	5.00	2.17	1.33	2.81	1.46	1.00	7.00

Abbreviations: CP, crossover point; FM, full‐membership; FNM, full non‐membership; BEC, benevolence ethical climate; HPE, high performance expectation; MA, Machiavellianism; OI, organizational identification; TL, transformational leadership; UPB, unethical pro‐organizational behaviors; SD, standard deviation.

## 5 Analysis and Results

### 5.1 Necessary Condition Test

In order to reveal the complex causal relationship between variables and outcomes, it is necessary to conduct a necessity condition test on the variables to determine if they must be presented when UPB occurs. According to the calculation rules of fsQCA, the necessity of individual variables needs to be assessed through consistency and coverage. Consistency reflects the extent to which the conditional variables are necessary for the outcome variable. The higher the consistency value, the higher the degree of necessity. When the consistency level exceeds 0.9, it indicates that the variable is a necessary condition for the outcome. Coverage is used to test the sufficiency of the conditional variables in explaining the outcome variable. Generally, when the coverage exceeds 0.8, it indicates that the variable is a sufficient condition for the outcome (Coduras et al. 2016).

One fundamental assumption of our configurational analysis is that no single variable alone, such as Machiavellianism, organizational identification, transformational leadership, high performance expectations, or ethical climate, can explain UPB. Instead, there are multiple configurations that are equally effective. This assumption is well supported as we conducted a necessity analysis on the antecedent variables using the fsQCA 4.1 software (see Table 3). The consistency of all five conditional variables with the outcome variable is less than 0.9, indicating that none of these variables is a single necessary condition for influencing UPB. In other words, all the conditional variables cannot individually determine the level of UPB. The interaction between specific individual and situational conditions seems to be complex, suggesting that these variables should be considered in a synergistic and simultaneous manner resulting in UPB.

**TABLE 3:** Necessary condition test.

Antecedent	UPB		~UPB	
	Consistency	Coverage	Consistency	Coverage
MA	0.773	0.795	0.434	0.451
~MA	0.467	0.447	0.803	0.782
OI	0.738	0.737	0.513	0.519
~OI	0.520	0.512	0.741	0.741
TL	0.758	0.750	0.498	0.498
~TL	0.494	0.491	0.751	0.759
HPE	0.693	0.676	0.565	0.558
~HPE	0.548	0.553	0.673	0.689
BEC	0.728	0.786	0.418	0.456
~BEC	0.496	0.456	0.804	0.750

*Note:* ~ means this condition/outcome does not exist. BEC, benevolence ethical climate; HPE, high performance expectation; MA, Machiavellianism; OI, organizational identification; TL, transformational leadership; UPB, unethical pro‐organizational behaviors; ~UPB, non‐unethical pro‐organizational behaviors.

### 5.2 Truth Table and Configuration Analysis

Configuration analysis is the core of the QCA method. It primarily examines whether the configurations formed by different antecedent conditions are subsets of the outcome set, in order to reveal the sufficiency of each configuration in explaining the outcome. Although consistency is also used to measure the sufficiency of configurations, the minimum threshold and calculation method are different from the necessity analysis. In general, a consistency level of no less than 0.75 is considered sufficient (Ragin 2008). However, the actual consistency thresholds used in existing literature vary; for example, 0.76 in Zhang and Du (2019), 0.8 in Du et al. (2021). Ragin (2008) suggested that the frequency threshold should be determined according to the sample size. For small to medium‐sized samples, the frequency threshold can be set at 1, while for datasets with more than 150 samples, the frequency threshold can be set at 3 or more, and the distribution of samples in the truth table should also be considered. Based on the above analysis, this study sets the case frequency threshold at 4 and the consistency threshold at 0.80, and sets the PRI (Proportional Reduction in Inconsistency) value at 0.70 to reduce the probability of configurations with consistent/inconsistent causal paths.

By analyzing the truth table, we can identify complex solutions, parsimonious solutions, and intermediate solutions. The core conditions for each solution can be identified through comparing the nesting relationship between complex and parsimonious solutions. The conditions that appear in both the complex and parsimonious solutions are considered core conditions for that solution, while conditions that only appear in the complex solution are considered marginal conditions (Ragin 2008). This study identifies five configuration patterns that can explain the occurrence of UPB (see Table 4). The overall consistency of these five configurations is 0.771, indicating that approximately 77% of the cases that satisfy these five configuration patterns exhibit high levels of UPB. The overall coverage is 0.779, indicating that these five configuration patterns can explain approximately 78% of the cases with high levels of UPB. The consistency and coverage of the configuration patterns derived in this study meet the standards defined by Ragin (2008) for consistency (> 0.75) and coverage (> 0.20), indicating the results of the configuration analysis on UPB are valid, and enabling further exploration of the driving mechanisms of UPB from individual and situational factors.

**TABLE 4:** Configurations for UPB and ~UPB.

Antecedent	UPB					~UPB				
	H1a	H1b	H1c	H2	H3	G1a	G2b	G2	G3	G4
MA			▲	△	●	◎	◎	◎	◎	◎
OI		▲	△	●				◎	◎	▲
TL	▲			●		△			▲	▲
HPE		▲	△		●		▲		▲	◎
BEC	●	●	●		△	◎	◎	◎		▲
Raw coverage	0.647	0.499	0.261	0.339	0.298	0.617	0.411	0.609	0.263	0.217
Unique coverage	0.099	0.014	0.016	0.296	0.038	0.015	0.012	0.018	0.013	0.027
Consistency	0.812	0.835	0.897	0.741	0.817	0.888	0.861	0.896	0.878	0.868
Solution coverage	0.779					0.716				
Solution consistency	0.771					0.847				

*Note:* ● indicate the presence of core condition, ◎ indicate the absence of core condition, ▲ indicate the presence of auxiliary condition, △ indicate the absence of auxiliary condition, spaces indicate the condition can be presence or absence, ~ indicate the condition/outcome does not exist. BEC, benevolence ethical climate; HPE, high performance expectation; MA, Machiavellianism; OI, organizational identification; TL, transformational leadership; UPB, unethical pro‐organizational behaviors; ~UPB, non‐unethical pro‐organizational behaviors.

#### 5.2.1 Configuration Analysis of UPB

**FIGURE 2:**
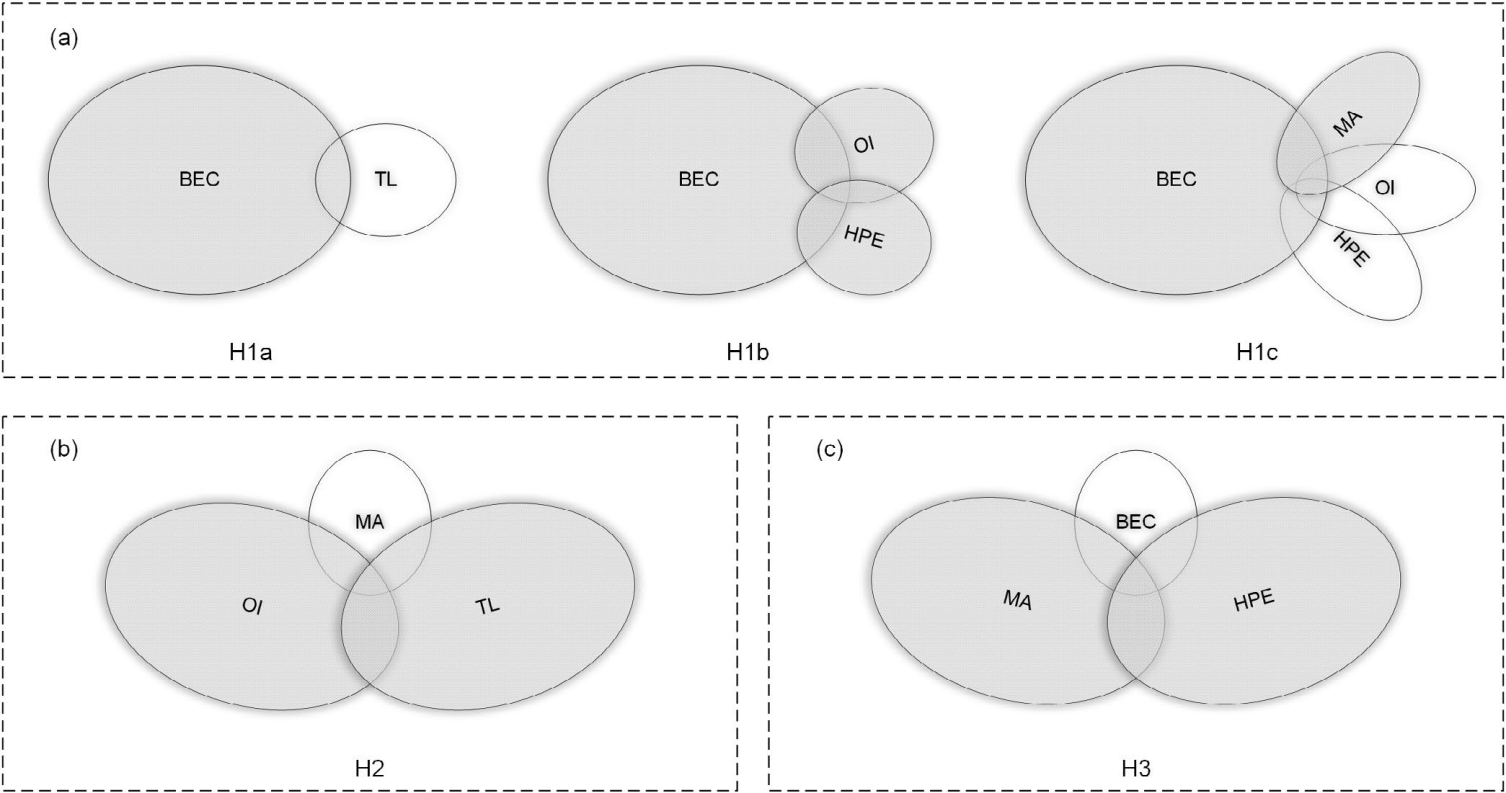
Configurations for UPB. (a) Configurations between BEC and one to three factors, (b) Configuration between OI, TL and MA, (c) Configuration between MA, HPE and BEC. BEC, benevolence ethical climate; HPE, high performance expectation; MA, Machiavellianism; OI, organizational identification; TL, transformational leadership; UPB, unethical pro‐organizational behaviors.

The complementarity between individual and situational conditions may ultimately influence the occurrence of employees' UPB. In our truth table results, the core conditions of paths H1a, H1b, and H1c are consistent and can be classified as the same parsimonious solution. Therefore, the five configurations can be summarized into three paths that affect UPB (see Figure 2). Figure 2 visually depicts three configurations that impact employees' UPB. Each configuration is represented by a combination of core and marginal conditions, which are indicated by different types of ovals (large gray solid ovals represent the presence of core conditions, large hollow ovals represent the absence of core conditions, small gray solid ovals represent the presence of auxiliary conditions, and small hollow ovals represent the absence of auxiliary conditions). These configurations are crucial for understanding the complex relationships between various factors and UPB.

*Configuration 1* (*H1a, H1b, H1c*): As shown in Figure 2a, in these configurations, the benevolence ethical climate is the core condition leading to employees' UPB. This indicates that employees' UPB depends primarily on the benevolence ethical climate, supplemented by other individual or situational conditions, corresponding to Configurations H1a, H1b, and H1c in the Table 4. Specifically, within these three detailed configurations, although they share similarities, there are differences in the marginal conditions. Configuration H1a (TL*BEC) (where “~” means “non”, “*” means “with”, the same below) suggests that under a benevolent ethical climate, if organizational leaders exhibit a transformational leadership style, regardless of other conditions, employees would be more willing to take risks and engage in UPB to reciprocate the leader (Yan and Zeng 2018). This configuration can explain approximately 64.7% of UPB. Configuration H1b (OI*HPE*BEC) suggests that under a benevolent ethical climate, employees are easily motivated to have a high level of identification with the organization. When the organization sets high performance requirements, employees will disregard unethical factors and engage in UPB (Yan et al. 2018). This configuration can explain approximately 49.9% of UPB. Configuration H1c (MA*~OI*~HPE*BEC) suggests that under a benevolent ethical climate, if the organization does not impose high performance expectations on employees with a high level of Machiavellianism, they will focus on maximizing their own interests, leading to a higher likelihood of engaging in UPB. This configuration can explain approximately 26.1% of UPB. Overall, Configuration 1 highlights the significant role of the benevolence ethical climate in promoting UPB under different combinations of other factors.

*Configuration 2* (*H2*): In the configuration as shown in Figure 2b (~MA*OI*TL), the core conditions for employees' UPB are organizational identification and transformational leadership, with the marginal condition being low Machiavellianism. This indicates that employees' UPB primarily depends on their identification with the organization and the leader's transformational tendencies, with low Machiavellianism playing a supporting role. This configuration can explain approximately 33.9% of UPB.

*Configuration 3* (*H3*): Figure 2c shows the configuration3 of MA*HPE*~BEC, the core conditions for employees' UPB are Machiavellianism and high‐performance expectations from the organization, with a low benevolence ethical climate as a marginal condition. This indicates that employees' UPB primarily depends on their Machiavellian tendencies and the organization's high‐performance expectations, supplemented by a low benevolence ethical climate. This configuration can explain approximately 29.8% of UPB.

#### 5.2.2 Configuration Analysis of Non‐UPB

There are five paths driving non‐UPB (see Table 4). Firstly, Configurations G1a (~MA*~TL*~BEC) and G1b (~MA*HPE*~BEC) show that non‐MA and non‐BEC play a core role, while non‐TL or non‐HPE play a supporting role, and employees do not engage in UPB. Secondly, Configuration G2 (~MA*~OI*~BEC) shows that non‐MA, non‐OI, and non‐BEC play a core role, leading to employees not engaging in UPB. Thirdly, Configuration G3 (~MA*~OI*TL*HPE) shows that non‐MA and non‐OI play a core role, while high transformational leadership and high‐performance expectations play a supporting role, leading to employees not engaging in UPB. Lastly, Configuration G4 (~MA*OI*TL*~HPE*BEC) shows that non‐MA and non‐HPE play a core role, while organizational identification, transformational leadership, and a benevolent ethical climate act as marginal conditions, leading to employees not engaging in UPB.

### 5.3 Sensitivity Analysis

Common methods for sensitivity analysis include adjusting calibration thresholds, changing case frequency, altering consistency thresholds, adding additional conditions, and supplementing or excluding cases (Schneider and Wagemann 2009). In this study, we conducted sensitivity analysis by adjusting consistency thresholds (changing the case truncation threshold from 4 to 6) and changing the measurement method of the outcome variable (replacing 90%, 50%, 10% with 95%, 50%, 5% as qualitative anchor points), The results were largely consistent, indicating the robustness of this study.

### 5.4 Predictive Validity Analysis

After assessing the robustness of the results through sensitivity analysis, we further evaluated the predictive validity of the model. The predictive ability of a complex causal model for the outcome variable can be explained by its predictive validity across different datasets. Obtaining a good fit for one particular model does not necessarily imply good predictive performance. To analyze predictive validity, it is necessary to examine whether the configurations obtained from one randomly truncated subsample of the original sample are highly applicable to another subsample in terms of consistency and coverage (Pappas and Woodside 2021), By doing so, we can evaluate the generalizability of the model across different subsets of the data.

We randomly divided the original sample into two subsamples and conducted a full fsQCA analysis on subsample 1. The configurations obtained from subsample 1 were consistent with the fsQCA results of the overall sample. The overall consistency (0.793) and overall coverage (0.724) met the standards (see Table 5). These values suggest that the configurations obtained from subsample 1 are highly consistent with the overall sample results, indicating a good fit and strong predictive power of the model. The good consistency and coverage of the configurations, further support the reliability of the model in predicting the outcome variable.

**TABLE 5:** Configurations for subsimple1.

Antecedent	UPB				
	N1a	N1b	N2	N3a	N3b
MA				●	●
OI	▲				
TL		▲	●	▲	
HPE	▲		●	●	●
BEC	●	●	△		△
Raw coverage	0.513	0.499	0.327	0.494	0.315
Unique coverage	0.057	0.081	0.023	0.033	0.030
Consistency	0.830	0.898	0.821	0.868	0.832
Solution coverage	0.724				
Solution consistency	0.793				

*Note:* ● indicate the presence of core condition, ◎ indicate the absence of core condition, ▲indicate the presence of auxiliary condition, △indicate the absence of auxiliary condition, spaces indicate the condition can be presence or absence. BEC, benevolence ethical climate; HPE, high performance expectation; MA, Machiavellianism; OI, organizational identification; TL, transformational leadership; UPB, unethical pro‐organizational behaviors.

**FIGURE 3:**
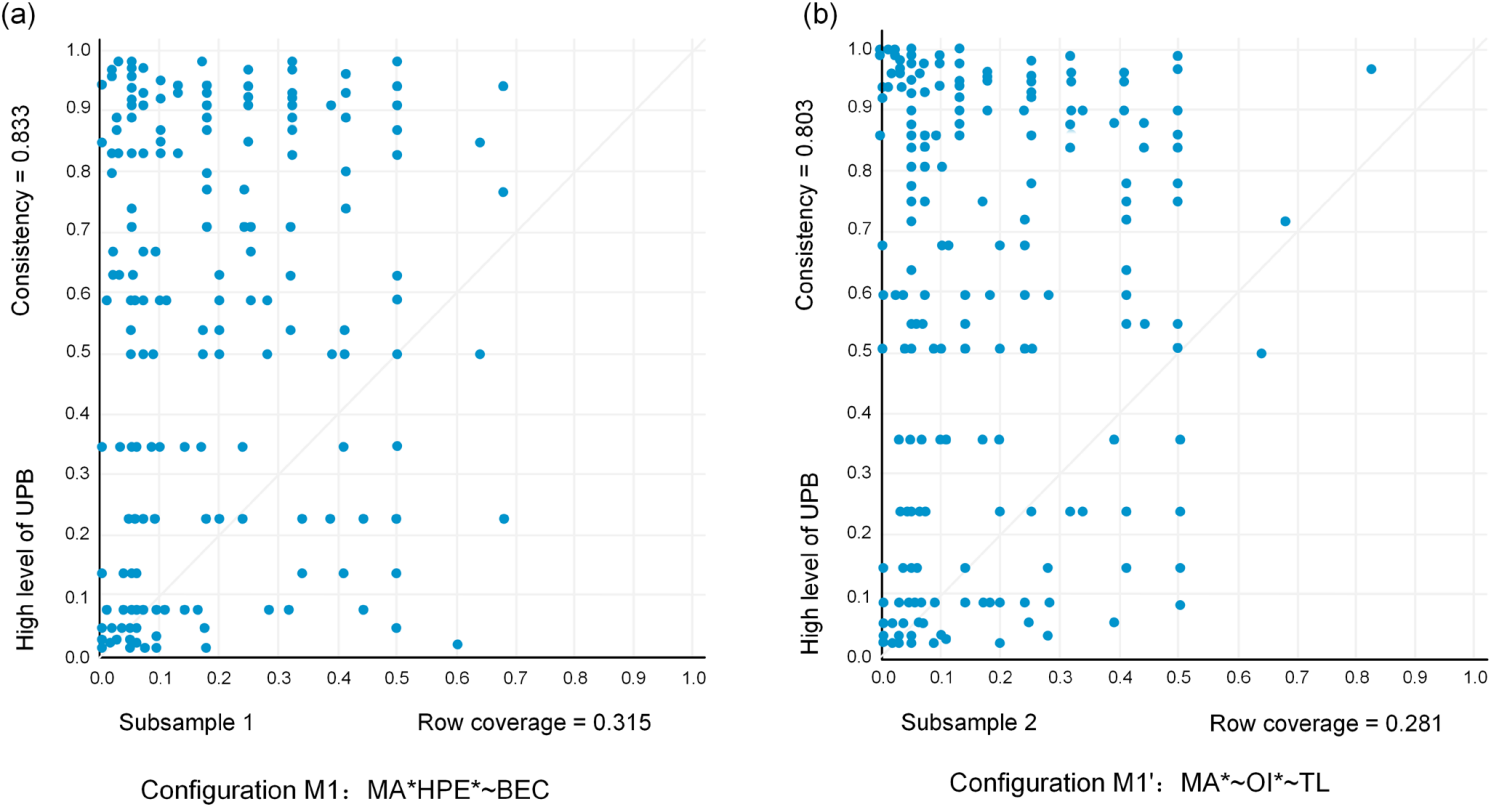
Configuration M1 (by subsample 1) and M1′ (by subsample 2). BEC, benevolence ethical climate; HPE, high performance expectation; MA, Machiavellianism; OI, organizational identification; TL, transformational leadership; UPB, unethical pro‐organizational behaviors.

As depicted in Figure 3, an XY plot visually illustrates the causal combinations within a model, effectively representing the relationship between the independent variable (X) and the dependent variable (Y). In this study, we selected the high–consistency configuration N3b from subsample 1. Then, we utilized the fuzzy–set “and” operation function in subsample 1 to create a new variable M1 (MA*HPE*~BEC). Subsequently, we compared M1 with the outcome variable (UPB) by using the “XY plot” option in the fsQCA 4.1 software, as shown in Figure 3a. The configuration model generated from subsample 1 was then applied to subsample 2. This application resulted in the creation of a new variable M1′ (MA*~OI*~TL). An XY plot was also generated in a similar manner, as presented in Figure 3b. The XY plots in Figure 3a,b demonstrated that the points representing the relationship between the variables were distributed in a similar pattern, with comparable consistency values and coverage values.

## 6 Discussion

### 6.1 The Driving Path of Benevolence Ethical Climate

In Configuration 1 (H1a, H1b, H1c), the benevolence ethical climate of the organization is to a certain extent the core driving force for employees' UPB. In the benevolence ethical climate, the interaction between the organization and employees forms an adaptive system, in which various factors influence each other and jointly shape employees' behaviors.

Firstly, the catalytic effect of transformational leadership. Based on the benevolence ethical climate, the transformational leadership style of the organization's leaders becomes a key driving factor for employees' UPB. Transformational leadership has characteristics such as personal charm, the ability to paint a beautiful vision for employees, and concern for employees' development needs. According to the social learning theory, employees will internalize the values of their leaders. Under the emotional bond created by the benevolence ethical climate, they are more inclined to transcend their immediate personal interests and pursue the long‐term interests of the organization. Further analysis from the perspective of the leader‐member exchange theory shows that under the benevolence ethical climate, transformational leadership can establish high‐quality exchange relationships with employees. By demonstrating personal charm and other behaviors, leaders make employees feel the particularity of their relationship with the leaders, so that employees transform their trust and gratitude toward the leaders into actions. Even in the face of moral dilemmas, they are more willing to take risks and engage in UPB to repay the leaders (Yan and Zeng 2018). For example, in some enterprises, leaders depict an extremely attractive corporate vision and at the same time give employees high‐level care, so that employees, in order to achieve corporate goals, do not hesitate to adopt some means that violate moral norms, such as fabricating data to improve performance indicators.

Secondly, the joint driving effect of organizational identification and high‐performance expectations. In the benevolence ethical climate, the organization emphasizes mutual assistance and love, has smooth emotional communication, fully implements the altruistic principle, and provides employees with comprehensive care and support. This positive organizational environment prompts employees to have a strong sense of organizational identification (Y. Zhang et al. 2017). Employees internalize organizational goals into personal goals, equate organizational interests with their own interests, and form a sense of “we” (Ashforth and Mael 1989). According to the self‐consistency theory, individuals have an internal need to maintain the consistency of their self‐concept. When the organization puts forward high‐performance requirements, employees who highly identify with the organization tend to ignore the unethical factors of their behavior in order to maintain an image consistent with the organization and safeguard their self‐cognition as “excellent organizational members”. For example, an Internet enterprise creates an atmosphere of caring for employees in its daily operations. The organization regularly holds team‐building activities and provides support for employees' career development planning, so that employees have a high level of identification with the enterprise. When the enterprise puts forward extremely high product development performance expectations to seize market share, some employees, in order to achieve the goals, may steal the trade secrets of competitors, plagiarize ideas, etc., believing that these behaviors are for the interests of the organization, and thus engage in UPB (Yan et al. 2018). At the same time, role theory points out that individuals will play specific roles in the organization and strive to meet the role expectations. Employees will position themselves as “members dedicated to the organization”, and high‐performance expectations become an important part of their role expectations. In order to meet this expectation, employees are more likely to break through the moral bottom line under the role of the emotional bond brought by the benevolence ethical climate.

Thirdly, the special choice of Machiavellian individuals. From the perspective of the personality‐situation interaction theory, an individual's personality traits interact with the situation they are in to influence their behavior. Under the benevolence ethical climate, when the organization does not impose high performance expectations on employees, the self‐centered personality traits of employees with high Machiavellianism dominate. They will focus on maximizing their own interests, and the possibility of engaging in UPB is greater. Specifically, such employees often take advantage of the organization's tolerance and trust toward employees in the benevolence ethical climate and put their personal interests above the overall interests of the organization. For example, in a creative design company, a designer with a high Machiavellian tendency, in the absence of high‐performance pressure, may leak the company's unreleased design plans to external enterprises in exchange for high rewards, claiming that it is to expand the company's business, but in fact to satisfy personal desires. Further analysis from the perspective of the moral disengagement theory shows that employees with high Machiavellianism are more likely to engage in moral disengagement. They will rationalize UPB through ways such as distorting the cognition of the behavior and shifting the responsibility. Under the benevolence ethical climate, such employees have more opportunities to find excuses and explain their self‐interested behavior as “trying new opportunities for the organization,” “promoting organizational change,” etc. For example, a marketing promotion staff member of an enterprise signed a high‐risk promotion agreement with an external partner without the company's authorization, claiming that it was to increase the company's market share, but in fact it was to obtain personal kickbacks from the partner. In addition, the self‐regulation mechanism in social cognitive theory also provides an explanation for this phenomenon. Under the benevolence ethical climate, the self‐regulation system of employees with high Machiavellianism tends to meet the need for maximizing personal interests. They will lower the moral standards for their own behavior and reduce self‐condemnation for unethical behavior, thus more easily engaging in UPB.

### 6.2 The Driving Path of Identification and Leadership Style

In Configuration 2 (H2), organizational identification and transformational leadership are the core conditions for employees' UPB. The specific analysis is as follows.

From the perspective of social identity theory, when employees have a high level of organizational identification, they will be willing to label their identities in the name of the organization, closely linking their personal reputation with the organization's reputation. On this basis, employees with low Machiavellianism often pay more attention to the harmony of interpersonal relationships and moral norms. They will generate an emotional response of repaying the organization based on their gratitude and loyalty to the organization. For example, in a well‐known technology enterprise, employees have developed a deep sense of identification with the enterprise through participating in corporate culture construction activities and receiving career development training. At the same time, these employees uphold relatively upright values (low Machiavellianism). When facing external competitive pressure, they will spontaneously safeguard the company's interests and may even unconsciously take some actions that go beyond the moral boundaries, such as stealing the business information of competitors to ensure the company's advantage.

Transformational leadership plays a key driving role in this configuration. According to the transformational leadership theory, the characteristics of transformational leaders enable leaders to fully mobilize the enthusiasm of employees and enhance employees' willingness to cooperate. Even if the organization does not put forward high performance expectations or lacks a benevolent ethical climate, transformational leadership can provide employees with strong emotional support through means such as emotional motivation and intellectual stimulation. From the perspective of the affective events theory, the positive emotional experiences that employees generate during the interaction with transformational leaders will further strengthen their commitment and loyalty to the organization. This emotional bond prompts employees to be more inclined to engage in UPB. Even in situations where moral judgment is ambiguous, in order to repay the organization and the leader, they will choose to engage in UPB. For example, the founder of a start‐up company has a highly transformational leadership style. He inspires employees' sense of mission by depicting the company's vision of becoming a leader in the industry in the future. At a crucial stage of the company's development, employees may take improper means to combat competitors in order to achieve this goal, believing that this is for the long‐term interests of the company and also a repayment for the leader's trust. In addition, from the perspective of the self‐consistency theory, employees' high level of organizational identification and loyalty to transformational leadership will closely bind their self‐concept with the organization's image. In order to maintain this consistency, employees will strive to practice behaviors that are in line with the organization's interests. When facing moral dilemmas, employees may rationalize unethical behaviors based on the perception of “being good for the organization”, thus reducing the internal moral conflict. At the same time, employees with low Machiavellianism are more inclined to follow social norms and moral principles in nature. However, under the influence of strong organizational identification and transformational leadership, their moral judgment criteria may shift, and they may regard UPB as a reasonable choice in special situations.

### 6.3 The Driving Path of Traits and Pressure

Configuration H3 reveals the deep logic of employees' behavioral choices under the interactive influence of the organizational environment and individual traits.

From the perspective of personality trait theory, employees with high Machiavellianism tend to regard others as tools to achieve personal goals and lack due respect and care for social moral principles and the interests of others. In an organizational environment with a low benevolence ethical climate, due to the lack of emotional support and moral guidance within the organization, the relationships between employees tend to be more utilitarian. This leads to a further amplification of the self‐interested tendencies of employees with high Machiavellianism (Sarwar and Song 2023). For example, in some highly competitive and cold sales teams, salespeople with high Machiavellianism may resort to any means to compete for customer resources for their personal performance, and even use unethical means such as slandering competitors and false advertising.

The organization's high‐performance expectations play a key catalytic role in this configuration. According to the stress cognitive theory, when employees perceive the high‐performance goals set by the organization, they will regard them as a source of stress. When employees with high Machiavellianism face this kind of stress, due to the lack of emotional connection with the organization and others, they are more inclined to transform the stress into the motivation to pursue personal interests. They will think that as long as they can achieve the goals and meet their own interests, it is reasonable to use unethical means. At the same time, McCall (1988) pointed out that when employees perceive high‐performance requirements, they will have a psychological burden, which in turn gives rise to a psychological suggestion that they cannot complete challenging work tasks. This psychological suggestion will prompt employees to ignore the consideration of the ethics of their behavior in order to get rid of the stress and achieve the goals. For example, in order to launch a new product in a short time, a technology company has set extremely high‐performance indicators for the R&D team. Members with high Machiavellianism in the team may steal the technological achievements of other teams or rush to release the product without sufficient product testing in order to meet the company's high‐performance requirements and seek promotion and rewards for themselves.

From the perspective of social learning theory, in an organization with a low benevolence ethical climate, there is a lack of positive moral models and guidance on behavioral norms. Employees with high Machiavellianism in such an environment cannot form correct moral cognition and behavioral patterns through observation and learning. On the contrary, they may be influenced by the surrounding utilitarian behaviors, further strengthening their tendency toward unethical behaviors. Moreover, since the organization does not provide sufficient emotional support and moral constraints, employees are more likely to cross the moral bottom line when facing high performance requirements. In addition, the moral disengagement theory can also explain this phenomenon. Employees with high Machiavellianism will rationalize UPB through means such as responsibility transfer and distortion of results. They may attribute their unethical behaviors to the organization's high performance requirements, thinking that “it is the company's pressure that forces me to do this”, thus reducing the internal moral condemnation and more wantonly engaging in UPB.

### 6.4 Causal Asymmetry of the Influence Paths of UPB and Non‐UPB

From the perspective of the paths influencing UPB, there are obvious differences between the configurations that lead to the occurrence of UPB and those that lead to the non‐occurrence of UPB. This difference reflects causal asymmetry; that is, the combinations of conditions leading to the occurrence and non‐occurrence of UPB are not simply opposite relationships, but have their own unique structures.

Based on social cognitive theory, the individual's cognitive process plays an important role in the causal asymmetry of UPB formation. Taking configuration H1c as an example, individuals with high Machiavellianism, in a benevolent ethical climate and when the organization has no high performance expectations for them, will start from the cognition of maximizing their own interests and are more likely to engage in UPB. In the configuration of non‐UPB, such as in G2, when an individual does not have Machiavellianism, organizational identification and a benevolent ethical climate, their cognition may be more inclined to abide by moral norms, so UPB does not occur. This shows that different cognitive tendencies of individuals will lead to different behavioral decisions, which in turn affect the occurrence of UPB.

Based on social exchange theory, social exchange relationships also have an impact on the causal asymmetry of UPB. In configuration H1a, in a benevolent ethical climate, when organizational leaders exhibit a transformational leadership style, employees, based on the psychology of social exchange, are more willing to take risks and engage in UPB in order to repay the leaders. This reflects the promoting effect of the social exchange relationship between employees and leaders on UPB. In the configuration of non‐UPB, such as in G4, although there are factors such as organizational identification, transformational leadership, and a benevolent ethical climate, due to the existence of core conditions such as non‐high performance expectations and non‐Machiavellianism, the social exchange relationship between employees and the organization may be in a state of balance, and UPB will not occur. Therefore, the social exchange relationship has different impacts on UPB under different combinations of conditions, which promotes causal asymmetry.

In addition, in configuration H3, the organization's high performance expectations and the individual's Machiavellianism serve as core conditions, and the low benevolence ethical climate serves as a peripheral condition, which may bring a certain situational pressure to employees, prompting them to ignore moral norms and engage in UPB in order to achieve high performance goals. In the configuration of non‐UPB, such as in G3, although there are high transformational leadership and high performance expectations, due to the existence of conditions such as non‐Machiavellianism and non‐organizational identification, this situational pressure may be alleviated, and employees will not engage in UPB. This indicates that the impact of situational pressure on UPB also varies under different combinations of conditions.

In conclusion, the causal asymmetry in the formation process of UPB is jointly driven by multiple factors. These factors interact with each other in different configurations, resulting in obvious differences in the combinations of conditions for the occurrence and non‐occurrence of UPB.

## 7 Conclusion

### 7.1 Main Conclusion

Based on the individual‐situation interaction theory, this study analyzes the multidimensional interaction effects of individual and situational factors on employees' UPB from the configurational perspective, and draws the following conclusions.

First, as a whole, factors such as Machiavellianism, organizational identification, transformational leadership, high‐performance expectations, and benevolence ethical climate cannot constitute the necessary conditions for employees' UPB alone. This indicates that employees' UPB is determined by multiple factors, and there is no single necessary condition. If we want to solve the problem of UPB in the organization, we need to work on many aspects. This includes employees' personality characteristics, perceptions and attitudes, organizational leadership styles, policies, and climate. By addressing these aspects comprehensively, we can reduce or eliminate UPB.

Second, sufficiency analysis shows that there are three paths driving UPB. These paths indicate that UPB is the result of the synergistic effect of multiple factors. The organization should fully consider and analyze various factors according to the actual situation and reduce the occurrence of UPB through different means. The configurations H1a, H1b and H1c proposed in this study all include the core condition: benevolence ethical climate, reflecting the important influence of BEC on UPB. As BEC strengthens, employees' UPB also increases accordingly, which is consistent with the conclusion of Zhang et al. (2017).

Third, there is asymmetric causality in the mechanisms driving UPB. The paths leading to high‐level UPB and non‐high‐level UPB are not entirely opposite. For instance, the presence of a transformational leadership style may contribute to high‐level UPB in one configuration, but its absence does not necessarily lead to non‐high‐level UPB in another. This indicates that the reasons for high‐level UPB cannot be simply reversed to explain non‐high‐level UPB.

### 7.2 Theoretical Contributions

Compared with other UPB‐related studies, this study's main theoretical contributions are as follows. One is to better understand the influencing factors of UPB, which further enhances the explanatory power of empirical research. Based on the individual‐situation interaction theory and existing literature, this study proposes an integrative analysis framework for the influencing factors of UPB. Previous studies have mostly focused on individual factors (Castille et al. 2018; Sarwar and Song 2023), or situational factors (Burnett 2017; Yan et al. 2018), and some have focused on how situational factors influence UPB through individual factors (M. Chen and Liang 2017; Y. Zhang et al. 2017). This study examined the effects of the interaction between individual and situational factors on UPB from a configuration perspective, which enriches the research context of the individual‐situation interaction theory. It also provides a more comprehensive understanding of the complex relationships between various factors and UPB, filling a gap in the existing literature.

The second is to better reveal the reasons for the occurrence of UPB, and further deepen the understanding of the complex mechanism behind UPB. Using a configuration perspective, this study empirically explores the synergistic effect of multiple conditions on UPB, identifying key combinations of factors that drive UPB. This expands the application of the configurational research in revealing UPB's causal complexity, answers the question of what factors determine UPB, and offers diversified research ideas. Compared with the latest research which has focused on factor‐by‐factor analysis, our study explores the non‐linear relationships among factors, providing a deeper understanding of the phenomenon.

### 7.3 Practical Implications

By exploring how individual and situational factors influence employees' UPB, this study offers practical guidance for organizational management.

First, it is critical for organizations to deeply understand the complex relationships among these key factors, pursue multiple‐factor synergy from a holistic perspective and select differentiated management paths accordingly. For large‐scale organizations, they may need to establish a comprehensive management system that integrates different departments to achieve the synergy among multiple factors. This could involve aligning recruitment strategies with leadership development programs and ethical climate initiatives. For small‐scale organizations, a more personalized approach may be needed, such as having regular one‐on‐one meetings with employees to understand their concerns and manage their behavior.

Second, organizations should advocate ethical values and guide employees to contribute to the organization in morally appropriate ways. While fostering a benevolent ethical climate, organizations need to be vigilant about potential risks. They should implement ethical training programs (e.g., using real case studies) to clarify ethical vs. unethical behavior.

Third, organizations should be cautious of the potential negative effects of transformational leaders. Leaders should lead by example and become moral exemplars for employees. When leaders' and employees' interests conflict with ethical norms (Castille et al. 2018), work ethics and ethical principles should be emphasized. Organizations should conduct regular ethical audits of leaders' behavior, and strengthen the moral assessment and work‐process supervision of leaders and employees to prevent employees from blind identification with the organization or leaders.

Fourth, organizations should pay attention to the personality traits of employees in recruitment and talent selection. They can use personality assessment tools to identify employees with high Machiavellian tendencies. For employees with such tendencies, organizations can provide targeted professional ethics training, such as workshops on ethical decision‐making in the workplace. This can guide them to self‐discipline at work (Yan and Zeng 2018).

### 7.4 Limitations and Future Work

Despite exploring UPB's multi‐factor synergy theoretically and practically, this study has limitations requiring further research. First of all, although the sample size meets configuration analysis requirements, it is relatively small for the large occupational groups. Future studies should expand the sample scale to enhance generalizability. Secondly, this study selected five antecedent conditions from the individual and situational aspects, but did not cover all of the UPB‐influencing factors (e.g., gender, psychological empowerment, superior‐subordinate relationship, and moral disengagement). Future research could use in‐depth interviews (with employees and managers) to identify additional factors, and integrate them to explore the full complex configuration of UPB drivers. Finally, although the theoretical model and the individual‐situation interaction theory can be well integrated and the research conclusions of this study further support the theory, other theoretical perspectives (e.g., AMO, TOE) could also help us gain a profound understanding of UPB. In future work, we plan to incorporate these theoretical perspectives into our research. We will compare the results obtained from different theoretical frameworks to gain a more comprehensive understanding of UPB and its underlying mechanisms.

## Ethics Statement

In accordance with the Declaration of Helsinki, the Bioethics Committee of Zhengzhou University of Light Industry (China) approved the research protocol (procedure number 20240112). Before the study began, all subjects received an oral and written description of the questionnaire survey and signed an informed consent form prior to participation. Following the initiation of the questionnaire survey, no substantial modifications were made to the methodology. The study adheres to the principles of openness, transparency, and reproducibility.

## Conflicts of Interest

The authors declare no conflicts of interest.

## Data Availability

The data that support the findings of this study are available from the corresponding author upon reasonable request.
